# The complete mitochondrial genome of *Aulacochilus grouvellei* Achard, 1923 (Coleoptera: Erotylidae: Erotylinae) with a phylogenetic analysis of Cucujoidea

**DOI:** 10.1080/23802359.2021.1901623

**Published:** 2021-03-23

**Authors:** Jing Liu, Weicheng Lu, Chenhao Lv, Josh Jenkins Shaw, Jing Li

**Affiliations:** a College of Plant Protection, Hebei Agricultural University, Baoding, PR China; b Key Laboratory of Zoological Systematics and Evolution, Institute of Zoology, Chinese Academy of Sciences, Beijing, PR China

**Keywords:** Mitochondrial genome, Erotylidae, *Aulacochilus grouvellei*, phylogeny

## Abstract

The complete mitochrondiral genome of *Aulacochilus grouvellei* Achard, 1923 was sequenced using an Illumina HiSeq platform. It represents the first mitochondrial genome of the subfamily Erotylinae. The mitogenome is a double-stranded circular molecule 15,607 bp in length with 22 transfer RNA genes, 13 protein-coding genes, 2 ribosomal RNA genes, and a control region. A Bayesian inference phylogenetic analysis including the new mitochondrial genome and a broad selection of other Cucujoidea recovered four major clades, including a ‘Cryptophagidae-Laemophloeidae-Cucujidae’ clade, a ‘Monotomidae-Nitidulidae’ clade, an Erotylidae clade, and a ‘Coccinellidae-Silvanidae’ clade. The family Erotylidae was recovered closely related to the ‘Cryptophagidae-Laemophloeidae-Cucujidae’ – ‘Monotomidae -Nitidulidae’ clade.

*Aulacochilus grouvellei* (Achard, 1923) is a typical species of Erotylinae, which is the largest subfamily of Erotylidae and mycophagous insects (Leschen 2003; Drilling et al. 2010). The species is easily recognized by each elytron bearing an orange ‘bird-shaped’ mark; pro, meso, and metacoxal lines present; terminal segment of maxillary palp length < 0.5 times as long as width. In this study, we sequenced and annotated the mitochondrial genome of *A. grouvellei*. It represents the first mitochondrial genome for the subfamily Erotylinae.

The specimen used for DNA extraction was collected by Xu Yan from Maomaoqing Village, Xishan District, Kunming City, Yunnan Province, China (N24.931841, E102.619698) and is deposited in Biological Control Laboratory of Hebei Agricultural University (Email: liujing15231147432@126.com and accession No.LJ003). Genomic DNA was extracted by DNeasy Blood & Tissue kit (QIAGEN, Germany). The Illumina HiSeq sequencing platform was used for high-throughput sequencing, and the sequencing read length was PE150. According to the principle of second-generation sequencing data, the original offline data was analyzed and the gene functions were annotated. The annotations of genes were done by Geneious version 8.0.5 software (Kearse et al. 2012), tRNAscan-SE version 1.21 (Schattner et al. 2005), and Mega version 7.0 (Sudhir et al. 2016).

The genome sequence data that support the findings of this study are openly available in GenBank of NCBI at (https://www.ncbi.nlm.nih.gov/) under the accession no. MW291531. The associated BioProject, SRA and Bio-Sample numbers are PRJNA689235, SRR13368788, and SAMN17204014, respectively. The complete mitochondrial genome (mitogenome) of *A. grouvellei* is a double-stranded circular molecule of 15,607 bp in length (GenBank accession number: MW291531), with 22 transfer RNA genes, 13 protein-coding genes (PCGs), 2 ribosomal RNA genes, and a control region. The overall base composition is A: 38.17%, T: 37.57%, C: 14.73%, and G: 9.53%, with a much higher A + T content. Among them, 23 genes are located on the mainchain (J) and 14 genes are located on the minority chain (N). The control region is 924 bp long and the base composition is 80.95% of A + T. Almost all of the 13 PCGs of *A. grouvellei* use the typical start codons ATN, except *ND1* uses TTG as the start codon. The 22 tRNAs can be folded into cloverleaf structures except *tRNA^Ser(AGN)^* whose dihydorouridine (DHU) arm simply forms a loop. *rrnL* is located between *tRNA^Leu (CUN)^* and *tRNA^Val^*, while *rrnS* is located between *tRNA^Val^* and the control region.

Phylogenetic analyses were conducted using MrBayes version 3.2 (Ronquist et al. 2012) with Bayesian posterior probabilities (PP) being caulculated based on the first and second codon positions of the PCGs and two rRNA genes. FigTree (Rambaut 2009) was used to visualize the phylogenetic tree. The ingroup taxa included ten species from Cucujoidea representing eight families and the outgroup taxa included *Cyclorhipidion bodoanus* (Curculionidae) and *Phyllobrotica quadrimaculata* (Chrysomelidae) due to their established close relationships to Cucujoidea (Bocak et al. 2014).

The phylogenetic tree (Figure 1) recovered four major clades, including a strongly supported ‘Cryptophagidae-Laemophloeidae-Cucujidae’ (PP = 0.99) clade and ‘Monotomidae-Nitidulidae’' clade (PP = 1). Erotylidae was recovered as sister to the two above clades with maximal support (PP = 1). The ‘Coccinellidae-Silvanidae’ clade was recovered with maximal support (PP = 1) and together they were sister to Cryptophagidae, Laemophloeidae, Cucujidae, Monotomidae, Nitidulidae, and Erotylidae (PP = 0.9). Our results are largely congruent with those of Robertson et al. (2015) in that the erotylid series are closely related to Monotomidae and Nitidulidae series. Increased taxon and mitogenome/genome sampling will be needed to better understand the relationships within Cucujoidea.

**Figure 1.**
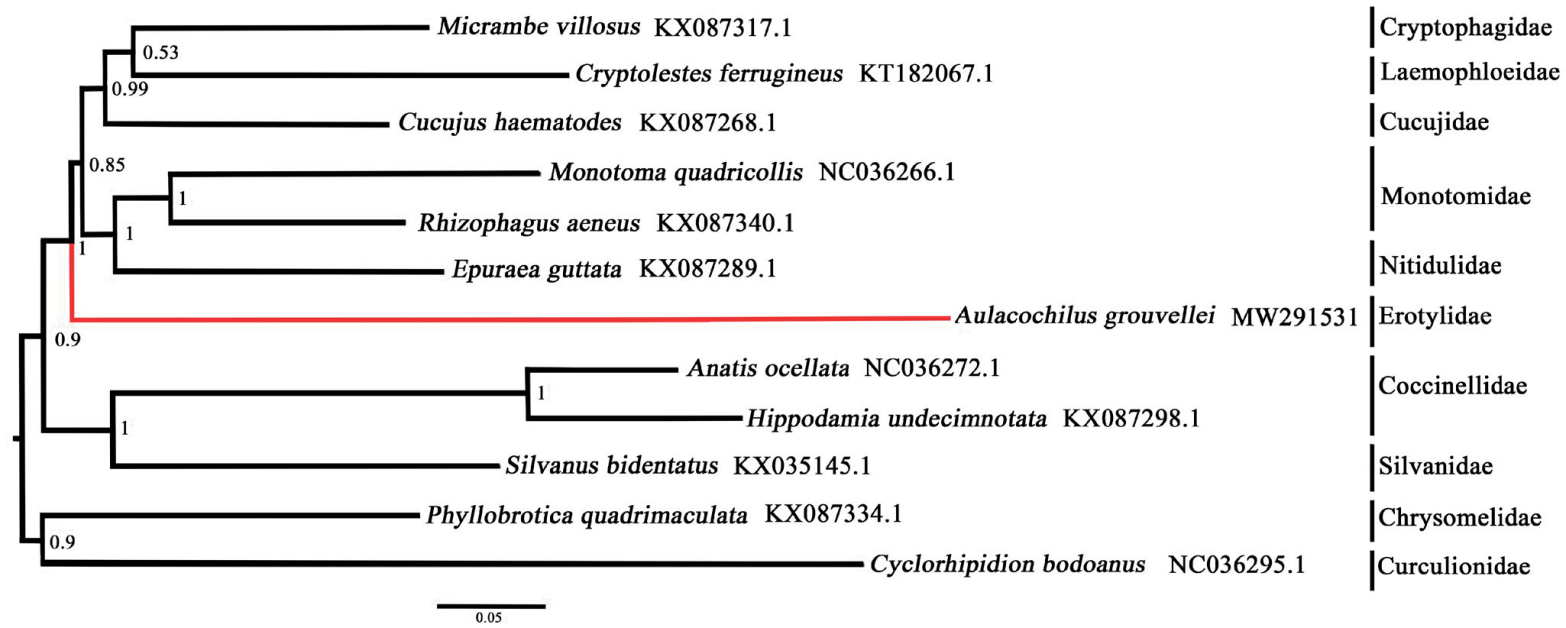
The Bayesian tree based on the PCG12R matrix (including PCGs first and second codons and two rRNAs) combined data sets. Numbers on nodes indicate Bayesian posterior probabilities. Red branch is the new data in this study.

## Data Availability

The genome sequence data that support the findings of this study are openly available in GenBank of NCBI at https://www.ncbi.nlm.nih.gov under the accession No. MW291531. The raw sequence data used in this research were deposited successfully with registered numbers of associated BioProject, SRA, and Bio-Sample: PRJNA689235, SRR13368788, and SAMN17204014, respectively.
